# Myopia Prevalence in Latin American Children and Adolescents: A Systematic Review and Meta-Analysis

**DOI:** 10.7759/cureus.63482

**Published:** 2024-06-29

**Authors:** Jaime Guedes, Alexandre B da Costa Neto, Bruno F Fernandes, Adriano C Faneli, Marcelo Alves Ferreira, Dillan Cunha Amaral, Denisse J Mora-Paez, Renato Ambrósio

**Affiliations:** 1 Ophthalmology, Glaucoma Research Center, Wills Eye Hospital, Philadelphia, USA; 2 Department of Ophthalmology, Federal University of the State of Rio de Janeiro, Rio de Janeiro, BRA; 3 Ophthalmology, Argumento Institute, Boucherville, CAN; 4 Medicine, Bahiana School of Public Health and Medicine, Salvador, BRA; 5 Statistics, São Paulo University, São Paulo, BRA; 6 Faculty of Medicine, Universidade Federal do Rio de Janeiro, Rio de Janeiro, BRA

**Keywords:** latin america, meta-analysis, myopia, prevalence, systematic review

## Abstract

Although myopia is a growing global concern, comprehensive studies on its prevalence among Latin American (LATAM) children and adolescents are still lacking. Thus, we conducted a systematic review and meta-analysis to determine the prevalence of myopia in LATAM children and adolescents aged three to 20. The study conducted a thorough literature search from January 1, 1975, to February 28, 2023, identifying 24 studies on the prevalence of myopia in LATAM that met the inclusion criteria. Quality assessment and standardized data collection were performed. The meta-analysis used a random-effects model due to heterogeneity and calculated prevalence rates. Finally, the analysis of data from 24 eligible studies revealed a myopia prevalence of 8.61% (range 0.80-47.36%, 95% confidence interval (CI): 5.22-13.87%, p < 0.05) among 165,721 LATAM children and adolescents. No significant age-based associations or temporal trends were observed in this study. Studies with non-cycloplegic or objective assessment exhibited a numerically higher, although statistically non-significant, myopia prevalence (10.62%, 95% CI: 4.9-21.6%) compared to studies using cycloplegia (7.17%, 95% CI: 3.40-14.50%). In conclusion, myopia affects approximately one in 11 LATAM children and adolescents. Given the increasing exposure of LATAM youth to known myopia risk factors, such as extensive near-work, online learning, and limited outdoor activities, it is crucial to monitor myopia trends in this region. Further research is imperative to address and prevent myopia in LATAM.

## Introduction and background

Uncorrected refractive errors are the leading cause of visual impairment, affecting over one billion individuals globally [1]. Myopia, the most common refractive error, affects school-aged children and young adults. The worldwide prevalence of myopia has surged to pandemic proportions, primarily due to lifestyle shifts and the widespread use of modern technology, notably mobile devices [2]. In 2001, myopia affected 22.9% of the global population, with projections indicating a 117% surge to 49.8% by 2050, impacting 4.8 billion individuals [2]. As of 2015, approximately 1.89 billion people worldwide had myopia, including 170 million with high myopia [3]. High myopia, typically defined as a spherical equivalent ≤-5.00 D [4-6], increases the risk of sight-threatening conditions, such as retinal holes, tears, degeneration, detachment, and myopic macular degeneration [3]. The escalating prevalence of myopia exerts a significant economic burden due to visual impairment and associated ocular issues, with the annual financial burden of refractive errors, including myopia, estimated at approximately $202 billion, surpassing the costs associated with other eye diseases [7].

In children aged five to 17 years, myopia prevalence rates vary considerably, ranging from 1.2% in Mechi Zone, Nepal, to 73.0% in South Korea [5,8]. Among the Chinese youth with a mean age of 18.5 ± 0.7 years, myopia prevalence increased from 79.5% to 87.7% over 15 years [9]. South African children aged five to 15 years have reported a myopia prevalence of 9.6% by the age of 15 [10]. The increasing prevalence of myopia has spurred research into its developmental mechanisms, revealing two primary factors: genetic influences (nature) and environmental effects, including lifestyle. Epidemiological data have provided substantial evidence of the influence of near-work activities on the development and progression of myopia, as children spend extensive hours engaged in such activities [11-14]. Certain ethnic groups seem more susceptible to similar environmental factors and cultural patterns, highlighting the need for further studies on geographical variations in the prevalence of myopia [15].

In Latin America (LATAM), urbanization has significantly altered the lifestyle and behavior of the population. The urban population has grown from 40 million in 1950 to 533 million in 2021 [16,17]. For example, urban areas in Brazil account for over 81% of the population [18]. Consequently, children and young adults in LATAM engage in more indoor and nearby work activities than the previous generations. To the best of our knowledge, no study has compiled data on the prevalence of myopia among children and adolescents in LATAM. Thus, we conducted a systematic review and meta-analysis of this theme.

## Review

Methods

Database Search and Eligibility Criteria for Inclusion

This meta-analysis followed the methodological recommendations of the Preferred Reporting Items for Systematic Reviews and Meta-Analyses (PRISMA) 2020 guidelines (PROSPERO registration number: CRD42023457987). We conducted a comprehensive search for studies on the prevalence of myopia among children and adolescents in LATAM using online data from three databases (Web of Science, SciELO, and PubMed). The searchers started on September 2023 and ended on December 2023. References from all the included studies, previous systematic reviews, and meta-analyses were manually searched for additional studies. Two authors (A.F. and M.F.) independently extracted data using predefined search criteria and quality assessment. The full articles of eligible publications were then scrutinized. This review included English, Spanish, and Portuguese studies published between 1975 and 2023 that focused on the prevalence of refractive errors in children and adolescents aged three to 20. Other inclusion criteria were observational cross-sectional studies that clearly described the sampling technique, specified the method of measuring refractive error (cycloplegic or non-cycloplegic refraction), used objective or subjective refraction, defined myopia based on a criterion of spherical equivalent ≤−0.5 D, and were either school-based or population-based.

The initial search terms were "refractive error AND children AND Latin America." In a subsequent search, the terms "prevalence" and "myopia" were used instead of "children" and "refractive error," respectively. Publications that included data from individuals with comorbidities, hospital populations, or uncertainties regarding age range delimitation were excluded.

Statistical Analysis and Risk-of-Bias Assessment

The meta-analysis was performed using R version 4.2.3 (2023-03-15 curt) "Shortstop Beagle" (R Foundation for Statistical Computing, Vienna, Austria). The "meta" package was utilized to generate forest plots illustrating the prevalence of myopia across individual studies, including their respective weights and the pooled prevalence with associated 95% confidence intervals (CIs) [19]. A funnel plot was used to assess potential bias and minor/significant study effects. Asymmetry was evaluated using the Begg's test [20]. The prevalence data were categorized into separate datasets based on cycloplegic or non-cycloplegic refraction and the objective or subjective methods employed to assess refractive error. The studies were classified into cycloplegic and objective refraction measures (complete method group) and non-cycloplegic or subjective refractive error evaluations (incomplete method group). A meta-regression model was employed to examine the potential variation in myopia prevalence with age, using the mean age reported in the studies. Another meta-regression model was used to explore the variation in the prevalence based on the year of data collection [21]. The heterogeneity test conducted across various studies revealed a substantial level of inconsistency (I^2^ = 99.8%), suggesting using a random-effects model to estimate the prevalence of myopia in LATAM children and adolescents in all meta-analyses. The original proportions were transformed into "logit" values (log(p/(1-p))), and the weights were calculated based on the inverse of the variance of proportions.

Results

Description of the Included Studies

**Figure 1:**
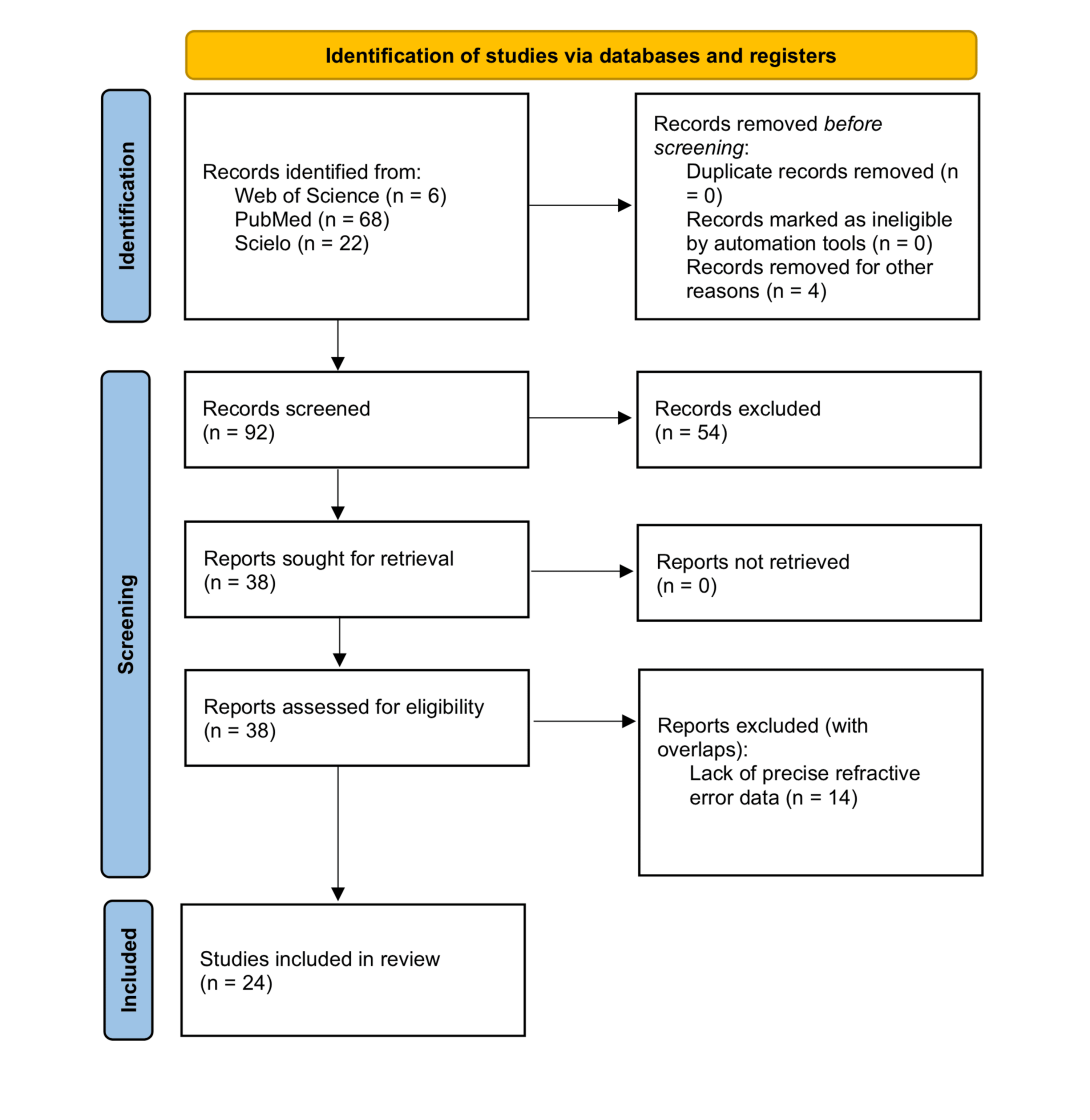
Preferred Reporting Items for Systematic Reviews and Meta-Analyses (PRISMA) 2020 flow diagram for new systematic reviews, which included searches of databases and registers only.

The searches in the three databases yielded six, 68, and 22 studies, respectively. Subsequently, the results were screened and filtered based on the source population, age limits, and specific myopia prevalence data availability. The final selection comprised publications that utilized general or school population data. We identified 24 studies in LATAM countries that assessed myopia prevalence (Table 1) [22-45]. Each study included in our analysis recorded information regarding the use of cycloplegia, the method of measuring refractive error (objective or subjective), the prevalence of myopia, and the corresponding sample size. The study inclusion flowchart is shown in Figure 1.

**Table 1:** Summary of the studies included in the analysis.

First author	Year	Age group (years)	Mean age (years)	Total sample size	Prevalence	Cyclopegia	Objective refraction	Quality score
Yotsukura [22]	2021	5 to 19	10.60 (2.90)	421	20.43	No	Yes	10
Salomão [23]	2008	11 to 14	12.54 (1.12)	2441	5.45	Yes	Yes	10
Lira [24]	2012	5 to 18	11.45 (4.04)	778	9.64	Yes	Yes	10
Garcia [25]	2004	5 to 20	-	974	13.04	Yes	No	8
Ibrahim [26]	2013	10 to 15	12.4 (1.60)	1590	3.14	Yes	No	10
Kara-José [27]	1975	7 to 13	9.38 (1.70)	1364	10.41	Yes	No	10
Ioschpe Gus [28]	2019		12.74 (3.31)	330	17.27	Yes	Yes	9
Schimiti [29]	1996	6 to 12	-	1966	8.24	Yes	Yes	9
Couto Jr [30]	2010		-	1800	1.06	Yes	Yes	9
Silva [31]	2015	3 to 7	4.5 (-)	2852	5.01	Yes	Yes	8
Estacia [32]	2004	6	7.10 (1.38)	88	10.23	Yes	Yes	10
Galvis 1 [33]	2014	8 to 17	11.4 (2.10)	1228	11.24	No	No	10
Lince-Rivera [34]	2016	2 to 14	-	112	1.79	No	No	9
Maul [35]	1998	5 to 15	9.56 (3.15)	5293	6.8	Yes	Yes	9
Villarreal [36]	1999	12 to 13	-	1035	44.25	Yes	Yes	9
Teran [37]	2019	15 to 18	-	3468	36.1	No	Yes	9
Verrone [38]	2007	6	6 (-)	177	1.69	Yes	Yes	9
Bastias [39]	2018	6 or 12	-	115598	47.36	No	No	8
Carter [40]	2005		-	476	0.84	Yes	Yes	9
Signes-Soler [41]	2019	5 to 14	9.1 (1.9)	2647	4.61	Yes	Yes	9
Galvis 2 [42]	2017	8 to 17	12.17 (2.63)	1933	11.59	No	Yes	10
Garcia-Lievanos [43]	2016	6 to 12	8.66 (1.90)	317	9.78	No	Yes	10
Rodriguez [44]	1993	5 to 14	-	17697	1.45	No	No	8
Rodriguez-Abrego [45]	2009	6 to 15	10.2 (2.43)	1136	33.01	Yes	Yes	10

Risk-of-Bias Assessment

**Figure 2:**
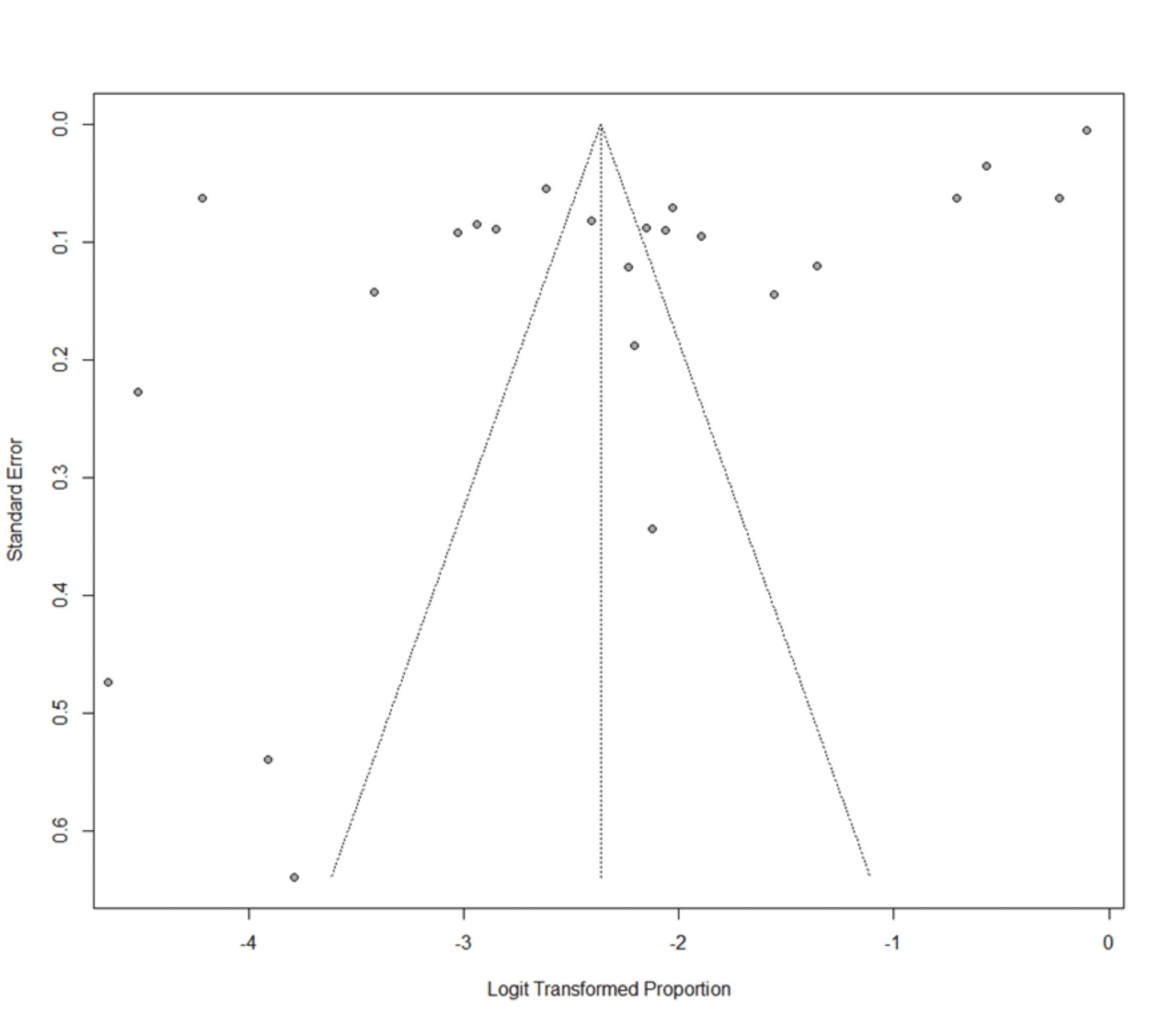
Funnel plot (risk-of-bias assessment).

The funnel plot and Begg's test for asymmetry showed homogeneity (z = 1.64; p = 0.1016), indicating that any potentially biased outliers did not significantly affect estimates (Figure 2).

Meta-Analyses

**Figure 3:**
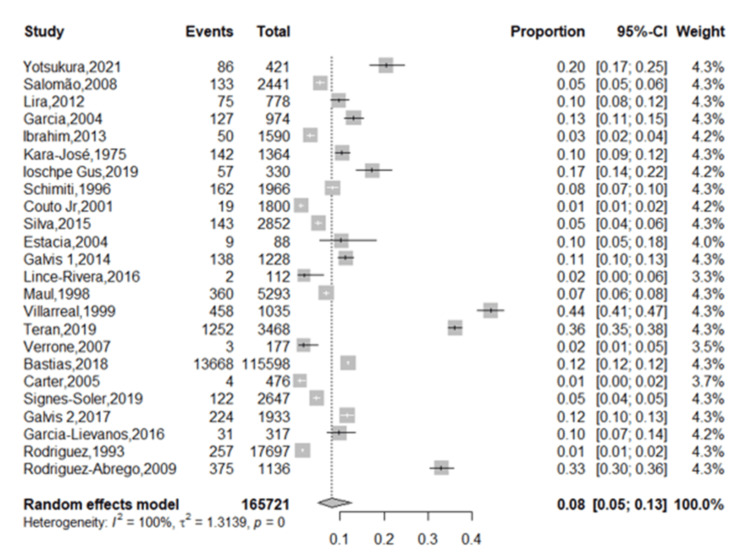
Forest plot illustrating the prevalence of myopia among Latin American schoolchildren aged five to 18. References: Yotsukura et al. [22], Salomão et al. [23], Lira et al. [24], Garcia et al. [25], Ibrahim et al. [26], Kara-José et al. [27], Ioschpe Gus et al. [28], Schimiti et al. [29], Couto Jr. et al. [30], Silva et al. [31], Estacia et al. [32], Galvis et al. [33], Lince-Rivera et al. [34], Maul et al. [35], Villarreal et al. [36], Teran et al. [37], Verrone et al. [38], Bastias et al. [39], Carter et al. [40], Signes-Soler et al. [41], Galvis et al. [42], Garcia-Lievanos et al. [43], Rodriguez et al. [44], Rodriguez-Abrego et al. [45]

**Figure 4:**
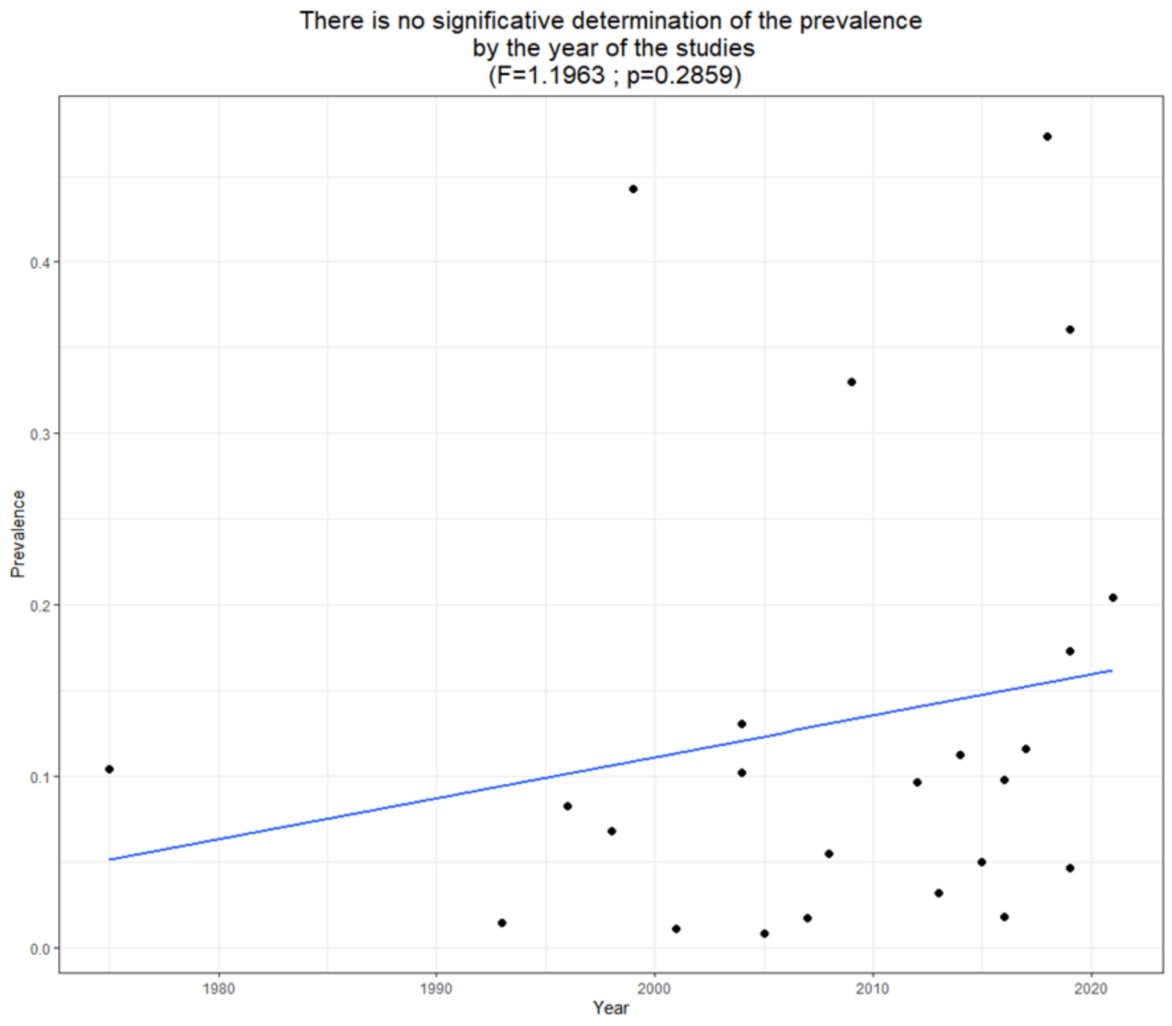
Meta-regression analysis indicated a positive trend between the percentage of myopia and the publishing year, although this association did not reach statistical significance.

**Figure 5:**
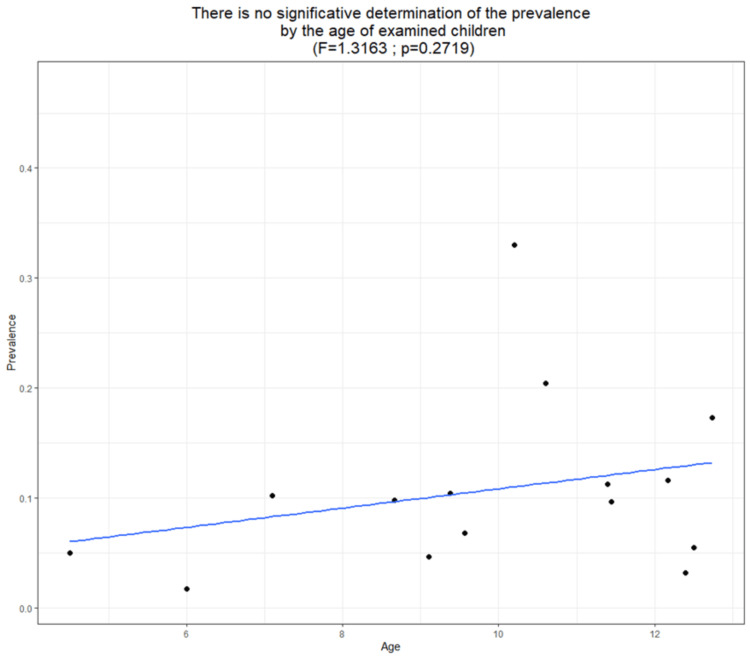
Meta-regression analysis revealed a positive trend between the percentage of myopia and the age of examined children, although this association did not reach statistical significance.

The sample size of children and adolescents aged three to 20 years in the study varied, ranging from 88 in a survey conducted in Brazil to 115,598 in one conducted in Chile. The estimated prevalence of myopia in LATAM was 8.61% (95% CI: 5.22, 13.87, p < 0.05) (Figure 3). The reported prevalence of myopia in these studies ranged from 0.80% to 47.36%. A meta-regression analysis of myopia revealed a trend for increased prevalence of myopia in more recent publications (Figure 4). However, this relationship was not statistically significant (p = 0.2859). In addition, meta-regression analysis of myopia based on the average age of the children demonstrated a trend for the prevalence of myopia to increase with age (Figure 5). Similarly, this relationship was not statistically significant (p = 0.2719).

Discussion

The present study analyzed 24 studies conducted over the past five decades. The prevalence of myopia, defined as a spherical equivalent refractive error (SER) ≤−0.5 D, in LATAM children and adolescents was 8.61% (95% CI, 5.22-13.87). No significant difference was observed in the prevalence of myopia according to the age of the children examined (p = 0.2719). The present study also demonstrated that cycloplegic refraction resulted in significantly lower estimates of myopia prevalence than non-cycloplegic refraction.

Overall, the reported prevalence of myopia in this meta-analysis was 46.52%. Compared to the results presented herein, previous studies have found a significantly higher prevalence, while others have found considerably lower rates [39,46]. Although differences in the definition of refractive error criteria are often suggested as a potential cause of variations in myopia prevalence across studies, this explanation may not apply to our research. This is because we specifically selected studies that defined myopia as a spherical equivalent of ≤−0.5 D. The relatively low prevalence of myopia observed in LATAM children and adolescents aligns with the findings from other studies, indicating a lower prevalence of myopia in Western children than in Asian children [8,47]. Numerous studies have demonstrated the significant influence of environmental factors on myopia development, particularly near-work activities, such as writing, reading, and computer use [48-50]. In many LATAM countries, there is a difference in the age at which children begin formal education compared with other Asian countries. In 2020, the preprimary school enrollment rate in Latin America was 77.5% [51], compared to 89.7% in China in 2012 [52]. This variance in educational practices results in young LATAM children being exposed to less near work and more engaged in outdoor activities, which could explain why LATAM children have a lower risk of developing myopia than their Asian counterparts. Nonetheless, a recent investigation has shown that more precise objective measures are required to draw definitive conclusions regarding the relationship between myopia and near work [53].

The prevalence of myopia remained statistically consistent across age groups. However, a slight increase in myopia prevalence was observed in the older age groups, suggesting an increasing trend in myopia prevalence with age. This observation is consistent with previous findings that reported a similar association between age and myopia prevalence [54,55]. The increased prevalence of myopia is believed to be linked to the increasing size of the eyeball as the child grows. The influence of sex on myopia prevalence has been inconsistent in the literature [56-59], with some studies proposing that the slightly higher prevalence in females may be related to variations in puberty onset between boys and girls. Other factors that could contribute to the higher prevalence of myopia in girls include less outdoor activity compared with boys [60].

The current study illustrated that cycloplegic refraction yielded notably lower estimates of myopia prevalence than non-cycloplegic refraction, in line with the literature [61-63]. Non-cycloplegic refraction overestimates myopia prevalence and produces unreliable measurements of myopia [64]. Therefore, cycloplegic refraction is considered the gold standard for myopia measurements [65]. Over half of the studies in this review employed cycloplegic refraction, which is particularly crucial in this age group where the difference between cycloplegic and non-cycloplegic refraction is significant [65]. Unfortunately, we could not demonstrate lower variability in the measured refractive error when using cycloplegic refraction than non-cycloplegic refraction. Non-cycloplegic refraction can be influenced by the variable accommodative state during examination, particularly in children of different ages with varying accommodation levels. This highlights the importance of appropriately controlling accommodation during refraction, particularly in young children who exhibit higher amplitudes of accommodation and more active accommodative responses [66,67].

This review had limitations that merit acknowledgment. First, the relatively limited number of published articles on the topic impeded our ability to establish a more definitive trend for the prevalence of myopia among LATAM children and adolescents. Second, studies that relied on subjective refraction and non-cycloplegic measures could have provided unreliable measurements of myopia. Third, we encountered a notable degree of heterogeneity across studies, which could have been caused by geographical differences or demographic characteristics of the sample. Nevertheless, we employed random-effects models to address and mitigate this limitation. Despite these limitations, this study addressed several crucial considerations. By including studies that consistently defined myopia as a spherical equivalent of ≤-0.5 D, we enhanced the comparability of reported prevalence rates. In addition, we excluded studies conducted on selected groups, such as hospital-based studies, and those lacking sampling evidence. Moreover, we assessed the robustness of each study design to ensure the reliability of the findings.

## Conclusions

In summary, this systematic review and meta-analysis highlighted a lower prevalence of myopia among LATAM children and adolescents than among Asian populations. It further emphasizes the importance of using cycloplegic refraction for the accurate and consistent estimation of myopia prevalence, as non-cycloplegic refraction can yield misleading results. Given the increasing exposure of the LATAM youth to known myopia risk factors, such as extensive near-work, online learning, and limited outdoor activities, it is crucial to monitor myopia trends in this region. Future research could investigate the impact of ethnicity on myopia prevalence, with the inclusion of various ethnic groups (Black, White, and Asian) to provide valuable insights into potential differences in myopia prevalence among these subgroups.
