# Correction: Myopia Prevalence in Latin American Children and Adolescents: A Systematic Review and Meta-Analysis

**DOI:** 10.7759/cureus.c349

**Published:** 2025-10-07

**Authors:** Jaime Guedes, Alexandre B da Costa Neto, Bruno F Fernandes, Adriano C Faneli, Marcelo Alves Ferreira, Dillan Cunha Amaral, Denisse J Mora-Paez, Renato Ambrósio

**Affiliations:** 1 Ophthalmology, Glaucoma Research Center, Wills Eye Hospital, Philadelphia, USA; 2 Department of Ophthalmology, Federal University of the State of Rio de Janeiro, Rio de Janeiro, BRA; 3 Ophthalmology, Argumento Institute, Boucherville, CAN; 4 Medicine, Bahiana School of Public Health and Medicine, Salvador, BRA; 5 Statistics, São Paulo University, São Paulo, BRA; 6 Faculty of Medicine, Universidade Federal do Rio de Janeiro, Rio de Janeiro, BRA

This article has been corrected to fix the following typos in the PRISMA diagram:

- 'Records identified from Web of Science' corrected from 9 to 6
- 'Records identified from PubMed' corrected from 9 to 68
- 'Records identified from Scielo' corrected from 24 to 22

